# Sex-Specific and Traumatic Brain Injury Effects on Dopamine Receptor Expression in the Hippocampus

**DOI:** 10.3390/ijms242216084

**Published:** 2023-11-08

**Authors:** Jaclyn Iannucci, Katherine O’Neill, Xuehua Wang, Sanjib Mukherjee, Jun Wang, Lee A. Shapiro

**Affiliations:** 1 Department of Neuroscience and Experimental Therapeutics, Texas A&M University School of Medicine, Bryan, TX 77807, USA; jmiannucci@tamu.edu (J.I.);; 2 Department of Biological Science, Texas A&M University, College Station, TX 77843, USA

**Keywords:** alcohol use disorder, dopamine, dopamine D2 receptor, mice, transgenic, neuroanatomy

## Abstract

Traumatic brain injury (TBI) is a major health concern. Each year, over 50 million individuals worldwide suffer from TBI, and this leads to a number of acute and chronic health issues. These include affective and cognitive impairment, as well as an increased risk of alcohol and drug use. The dopaminergic system, a key component of reward circuitry, has been linked to alcohol and other substance use disorders, and previous research indicates that TBI can induce plasticity within this system. Understanding how TBI modifies the dopaminergic system may offer insights into the heightened substance use and reward-seeking behavior following TBI. The hippocampus, a critical component of the reward circuit, is responsible for encoding and integrating the spatial and salient aspects of rewarding stimuli. This study explored TBI-related changes in neuronal D2 receptor expression within the hippocampus, examining the hypothesis that sex differences exist in both baseline hippocampal D2 receptor expression and its response to TBI. Utilizing D2-expressing tdTomato transgenic male and female mice, we implemented either a sham injury or the lateral fluid percussion injury (FPI) model of TBI and subsequently performed a region-specific quantification of D2 expression in the hippocampus. The results show that male mice exhibit higher baseline hippocampal D2 expression compared to female mice. Additionally, there was a significant interaction effect between sex and injury on the expression of D2 in the hippocampus, particularly in regions of the dentate gyrus. Furthermore, TBI led to significant reductions in hippocampal D2 expression in male mice, while female mice remained mostly unaffected. These results suggest that hippocampal D2 expression varies between male and female mice, with the female dopaminergic system demonstrating less susceptibility to TBI-induced plasticity.

## 1. Introduction

Traumatic brain injury (TBI) occurs in over 50 million people globally each year [1,2]. TBI is a major contributor to acute and chronic health problems, including affective disorders, neurological deficits, physical disabilities, and other comorbidities. After TBI, there may also be an increased risk for alcohol and drug consumption and abuse [3,4,5], suggesting that TBI may induce brain structural changes that functionally increase drug and alcohol-seeking behaviors.

Alcohol abuse, other substance use disorders, and risk-taking behaviors are all linked to the dopaminergic system, a major component of the reward circuitry in the brain [6,7,8,9,10,11,12,13]. The dopaminergic circuitry provides crucial connections between reward, motivation, and memory formation, and the anatomical pathways underlying the connectomic role in drug and reward-seeking behaviors continue to be elucidated [14,15,16,17,18]. Considering that TBI changes the dopaminergic system [19,20], elucidating specific dopaminergic components that are modified by TBI could provide valuable insight into the circuit alterations that might underlie the increased reward-seeking behavior after TBI.

The hippocampus plays a major role in reward behavior. One function of the hippocampus within the reward circuit is to encode and integrate the spatial and salient components of a rewarding experience [21,22,23,24]. Conditioned place preference (CPP), a behavioral phenomenon commonly used to study drug abuse in rodents, can be linked to signaling through numerous dopamine receptors in the hippocampus [18,25,26,27]. Importantly, TBI alters drug-induced CPP [28,29]. Sex differences in CPP, including after TBI, have also been identified [30,31,32]. Thus, it is important to describe both biological sex- and TBI-related effects on dopaminergic circuitry in the hippocampus.

Dopamine D2-like receptors (D2R) have been shown to influence synaptic plasticity and hippocampal function, including learning and memory [33,34]. D2 receptor expression in the hippocampus correlates with memory performance in adult males [35], and high levels of D2 receptor mRNA have been identified in the hippocampal dentate gyrus [36]. Numerous other studies identify hippocampal D2 expression in humans and rodents [18,33,37,38]. However, the full expression profile of D2 receptors in the hippocampus, including after TBI, has not been elucidated. Therefore, the current study tested the hypothesis that TBI causes alterations to neuronal D2 receptor expression in the hippocampus.

This study was also designed to test the hypothesis that there are sex differences in hippocampal D2 receptor expression in response to TBI. Sex-associated differences in D2 receptor expression have been previously identified, but these studies have not focused on the hippocampus [39]. D2-expressing tdTomato transgenic male and female mice were subjected to either a sham injury or our fluid percussion injury (FPI) model of TBI [40,41], followed by quantification of D2-expression in the various hippocampal subregions. The results indicate region-specific sex-related differences in D2 receptor expression in the hippocampus and demonstrate significant interactions between TBI-associated alterations to D2-expressing neurons and biological sex.

## 2. Results

### 2.1. Male and Female Mice Have Qualitative Differences in D2-Expressing Neurons in the Hippocampus

**Figure 1:**
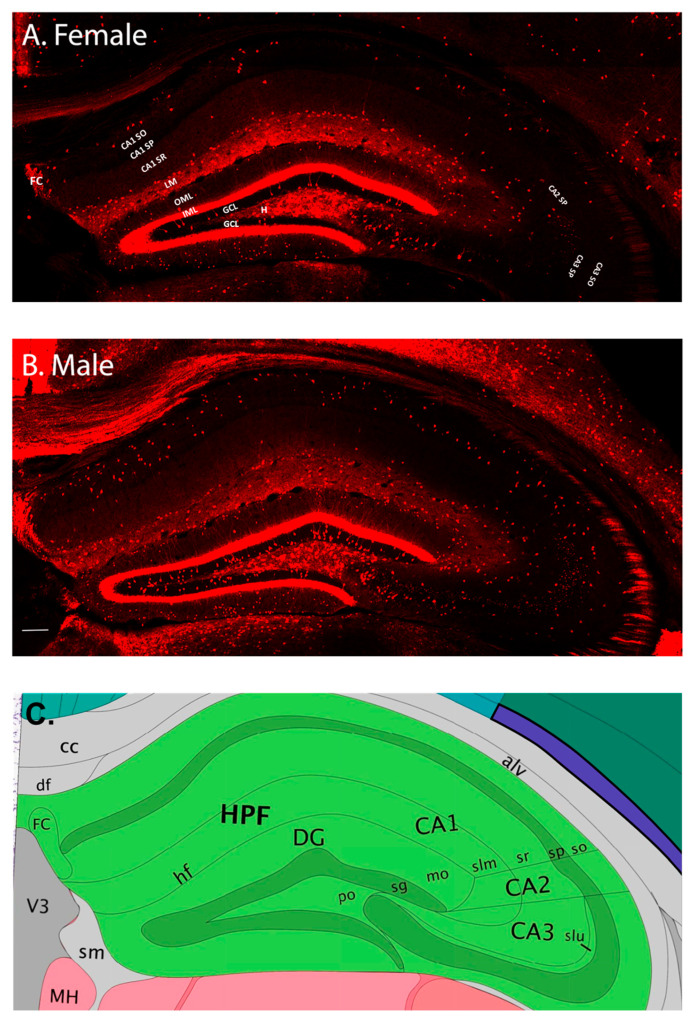
Qualitative analysis of hippocampal D2 receptor-expressing neurons in male and female mice. Representative confocal z-stack micrographs of a 10-week-old female sham (**A**) and male sham (**B**) hippocampus illustrate baseline D2 expression. Qualitative evaluation revealed differences in D2 expression patterns in females compared to age-matched males. These differences include a generally lower D2 expression in females compared to males, most visibly in the C3 and CA2 boutons, the granule cell layer (GCL), and the hilus. In the outer plexiform molecular layer of the suprapyramidal blade in male mice, D2-expressing cells are preferentially observed close to the border with stratum lacunosum moleculare (LM). However, in the female mice, the D2-expressing cells in this region exhibit a more heterogeneous expression. Additionally, patches of intense D2-expressing apical dendrites in the molecular layer (ML) of the dentate gyrus were observed in male mice that were less prominent in female mice. This may indicate a difference in the types of cells (e.g., D2-expressing vs. non-D2-expressing) being innervated by the perforant pathway. In the male mice, the CA3 and CA2 regions contain numerous small puncta-like stainings that likely represent D2-labeled mossy fiber boutons. These boutons are only sparsely observed in female mice. Female mice also exhibit lower D2 expression in the fasciola cinereum (FC). In this region, large D2-expressing cells and proximal portions of their processes were observed in both male and female mice, with the male mice appearing to have greater expression. In (**C**), a representative atlas image (Allen Mouse Brain Atlas, mouse.brain-map.org and atlas.brain-map.org) depicts hippocampal subregions (in green) in the mouse. Scale bar in (**B**) = 75 µm for both images.

The results of this study indicate that there are baseline differences in D2-expressing neurons in the hippocampus between male and female mice (Figure 1A,B). First, qualitative evaluation revealed changes in D2 expression patterns in females as compared to age-matched males. In the dentate gyrus of male mice, the D2-expressing cells in the granule cell layer appear to be much more abundant than in female mice (Figure 1). In the outer plexiform molecular layer of the suprapyramidal blade, D2-expressing cells are preferentially observed close to the border with stratum lacunosum moleculare. However, in the female mice, the D2-expressing cells in the suprapyramidal molecular layer exhibit a more heterogeneous expression, with cells frequently observed close to the inner plexiform layer (Figure 1). The suprapyramidal blade develops earlier than the infrapyramidal blade [42]. Cells born during embryonic development contribute preferentially to the outer granule cell layer (GCL), and cells born postnatally remain in the inner GCL, closer to the hilus [43,44,45,46,47]. Throughout our analysis in both male and female mice, we noticed patches of intense D2-expressing apical dendrites in the molecular layer of the dentate gyrus and other areas of sparse staining (Figure 1). It is possible that these D2-labeled dendrites received specific types of perforant pathway projections from the entorhinal cortex or other regions. In the male mice, evaluation of the CA3 and CA2 regions reveals small puncta-like staining that may represent D2-labeled mossy fiber boutons. These boutons are only sparsely observed in female mice. In addition to the mossy fiber pathway that prominently projects from the dentate granule cells to CA3, hippocampal CA2 receives a number of inputs, including from the hypothalamus [48], the dentate mossy fibers [49], and the entorhinal cortex, the latter of which contributes to the role of CA2 in social memory [50]. The fasciola cinereum (FC) is a curved midline continuation of the septal tip of hippocampal CA1 [51,52]. Large D2-expressing cells and proximal portions of their processes were observed in both male and female mice, with the male mice appearing to have greater expression in this region. Although not widely studied [53], the FC contains a heterogeneous cell population [54,55,56], receives inputs from the lateral entorhinal cortex [57], and is involved in the acquisition of visual contextual memory [53]. The different distributions of D2-expression in these regions suggest their potential involvement in sexually dimorphic differences in visual, spatial, and social memory processing.

### 2.2. Quantitative Differences between Male and Female D2-Receptor Expression in the Hippocampus

**Figure 2:**
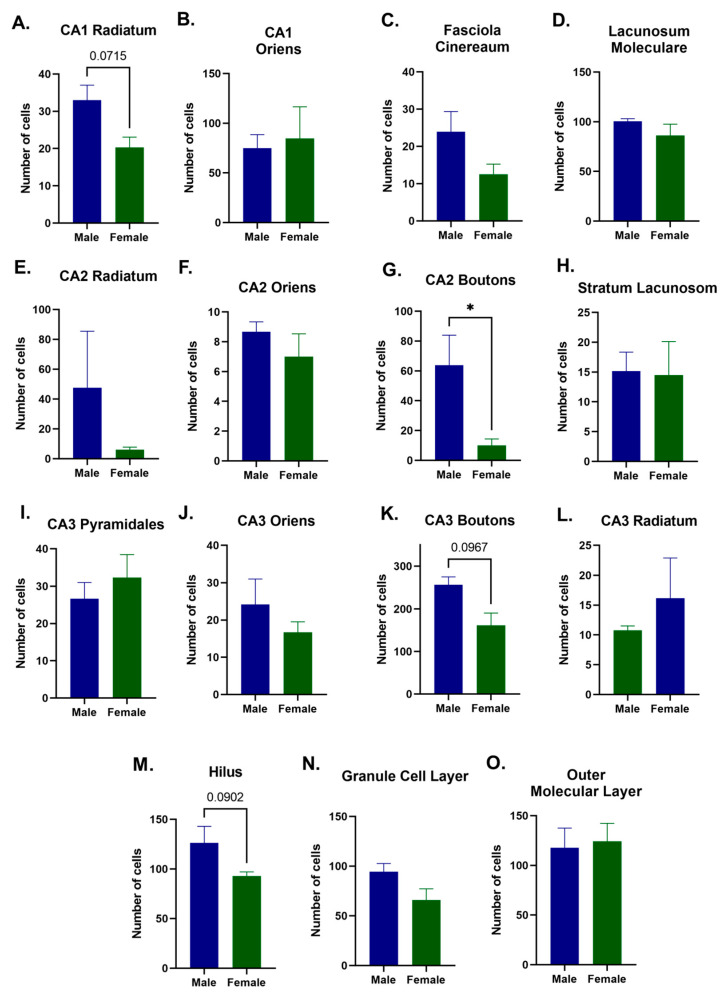
Sex differences in hippocampus D2 expression. Quantification of D2-expressing neurons by region of the hippocampus revealed modest baseline sex-related expression differences. In general, female mice trended towards less D2 expression compared to males. Female mice had lower D2 expression in the stratum radiatum of the CA1 (*p* = 0.0715, NS) (**A**). Female mice also had lower expression in both the CA2 (*p* < 0.05) (**G**) and CA3 boutons (*p* = 0.0967, NS) (**K**). This can also be seen in the hilus of the dentate gyrus (*p* = 0.0902, NS) (**M**), the granule cell layer (*p* = 0.1729, NS) (**N**), and in the fasciola cinereum (*p* = 0.1238, NS) (**C**). On the other hand, no significant changes or trends were observed in CA1 stratum oriens (**B**), lacunosum moleculare (**D**), CA2 stratum radiatum (**E**), CA2 stratum oriens (**F**), stratum lacunosum (**H**), CA3 stratum pyramidales (**I**), CA3 stratum oriens (**J**), CA3 stratum radiatum (**L**), and the outer molecular layer (**O**). Data expressed as mean ± SEM, n = 2 for males, n = 3 for females; * *p* < 0.05.

To further evaluate the extent of region-specific differences in hippocampal D2 expression, a quantitative analysis of each hippocampal subfield was performed. Consistent with the qualitative analysis, quantitative analysis of the small bouton-like D2-expressing elements in CA2 and CA3 revealed there were significantly fewer in CA2 (*p* < 0.05; Figure 2G) and a trend towards less in CA3 (*p* = 0.0967, NS; Figure 2K) of female mice. Trends that approached significantly lower D2 expression in females were also identified in the CA1 stratum radiatum (*p* = 0.0715, NS; Figure 2A) and in the dentate gyrus hilus (*p* = 0.0902, NS; Figure 2M). No other quantitative changes were identified between male and female sham mice in any of the other hippocampal regions examined. It is pertinent to note that in the dentate gyrus, the robustly labeled D2-expression in the inner molecular layer was only qualitatively assessed because the density of D2-expression precluded the necessary resolution for quantitative analysis.

### 2.3. Interactions between Sex and TBI on D2-Receptor Expression in the Hippocampus

Interactions between sex and injury in the hemisphere ipsilateral to the FPI were examined and are summarized in Table 1. Two-way ANOVA revealed a significant (*p* < 0.01) main effect of sex on D2 expression in the outer molecular layer (OML) and a significant interaction effect (sex × FPI) in the hilus (*p* < 0.01), GCL (*p* < 0.01), and OML (*p* < 0.05). Post hoc analysis revealed that the number of D2-expressing neurons was significantly greater for female mice that received FPI compared to their male counterparts in the hilus (*p* < 0.05), OML (*p* < 0.001), and GCL (*p* < 0.05), indicating a region-specific reduction in the male mice that received FPI. In hippocampal areas, CA3, CA2, and CA1, a significant main effect for sex was observed in CA1 stratum radiatum (*p* < 0.05) and CA2 axonal boutons (*p* < 0.05), and a significant interaction was observed in CA2 axonal boutons (*p* < 0.05) (Table 1).

A two-way ANOVA was also used to assess the hemisphere contralateral to the FPI (Table 2). The sex and interaction effects were primarily limited to the ipsilateral hemisphere, but there was a significant main effect of sex in the OML (*p* < 0.05), CA1 stratum oriens (*p* < 0.05), and CA3 stratum pyramidales (*p* < 0.05) of the contralateral hemisphere. There were no significant interaction effects identified in the contralateral hemisphere (Table 2). It should be noted that this analysis occurred 30 days post-FPI, and it is possible that at more acute, or more chronic, post-FPI time points, the changes to the contralateral hemisphere may be more pronounced.

### 2.4. FPI Reduces Neuronal D2 Expression in the Hippocampus of Male Mice

**Figure 3:**
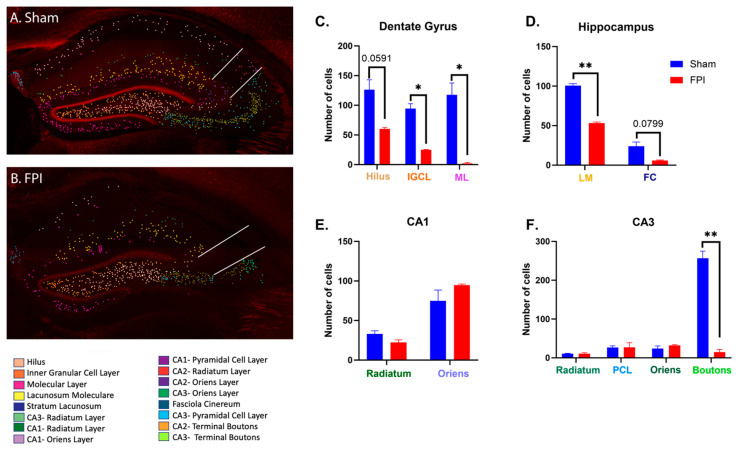
FPI reduces the expression of D2 in the male hippocampus. Representative confocal micrographs of a sham (**A**) and FPI (**B**) mouse show the overall decrease in D2-expression following FPI. In (**C**), quantitative analysis of the dentate gyrus shows reduced D2 expression in the hilus, granule cell layer (GCL), and outer molecular layer (OML). In (**D**), FPI significantly reduced D2 expression in the lacunosum moleculare (LM) and reduced expression in the fasciola cinereum (FC). In (**E**), FPI did not affect D2 expression in either CA1 stratum radiatum (SR) or stratum oriens (SO). In (**F**), FPI did not have any effect on the expression of D2 in the CA3 SR, stratum pyramidales (SP), or SO but did significantly reduce expression in the CA3 axonal boutons (AB). White lines in (**A**,**B**) delineate the boundary between CA3 and CA2 on the bottom and CA2 and CA1 on the top. Data expressed as mean ± SEM, n = 2; * *p* < 0.05, ** *p* < 0.01.

Further examination of D2 expression in the hippocampus of male mice revealed several significant changes in subregions of the ipsilateral hippocampus and dentate gyrus following FPI (Figure 3). In the dentate gyrus, there was a significant decrease in D2-expressing neurons in both the GCL (*p* < 0.05) and OML (*p* < 0.05) and a trend (*p* = 0.0591, NS) towards a decrease in D2-expressing neurons in the hilus after FPI (Figure 3C). There was also a significant loss of D2-expressing neurons in the lacunosum moleculare (*p* < 0.01; Figure 3D) in the male FPI mice when compared to sham controls (Figure 3E). In CA3, the D2-expressing axonal boutons were significantly decreased following FPI (*p* < 0.01; Figure 3F). There was a significant decrease in the CA1 stratum pyramidales (*p* < 0.05; not shown), but no significant reduction in the number of D2-expressing neurons found in other regions of the contralateral hippocampus. While these findings highlight the effect of FPI in reducing D2 expression in the hippocampus, it is important to recognize that these studies were conducted with a low n. It is also pertinent to note that sham mice may differ from intact mice, and results should be interpreted with these considerations. 

### 2.5. FPI Does Not Significantly Alter D2 Expression in the Hippocampus of Female Mice

**Figure 4:**
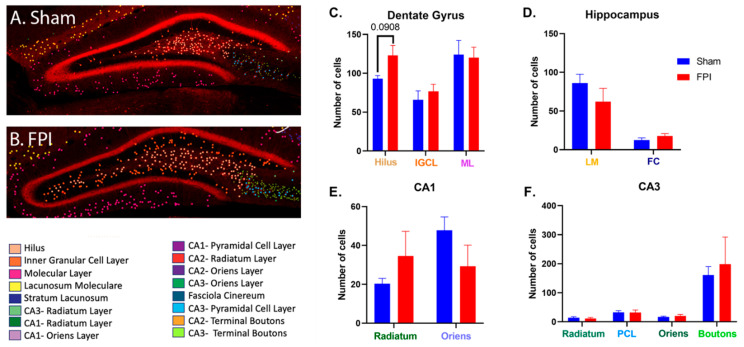
FPI does not significantly alter the expression of D2 in the female hippocampus. Representative confocal micrographs of a sham (**A**) and FPI (**B**) mouse illustrate the lack of a significant effect of FPI. In (**C**), no significant differences were observed in the dentate gyrus, including the granule cell layer (GCL) and outer molecular layer (OML). Interestingly, there was a trend towards increased expression in the hilus (*p* = 0.0908, NS). In (**D**), no significant changes were identified in the lacunosum moleculare (LM) or fasciola cinereum (FC). In (**E**), no significant differences were identified in sham versus FPI in the CA1 stratum radiatum (SR) or stratum oriens (SO). There were also no significant differences found in CA3 SR, SP, SO, or the CA3 axonal boutons (AB) (**F**). Data expressed as mean ± SEM, n = 3.

Previous studies examining TBI in female mice have shown that they are generally more resistant to injury-induced neurodegeneration [58,59,60]. Consistent with this notion, the female FPI mice did not have any significant effects on the D2-expressing neurons in either the ipsilateral or contralateral hemispheres (Figure 4A–F), compared to female sham mice. In the dentate gyrus, no significant effects of FPI on the D2-expressing neurons were observed, although a trend (*p* = 0.091, NS) towards an increase was observed in the ipsilateral hilus (Figure 4C).

## 3. Discussion

The results from the current study illustrate differences between male and female mice in hippocampal neuronal D2-expression. Compared to male sham mice, female sham mice exhibited fewer D2-expressing axonal boutons in both the CA3 and CA2 stratum radiatum. Fewer D2-expressing neurons were also observed in the hilus and in the CA1 stratum radiatum of female sham mice. These findings are consistent with previous descriptive studies that examined D2 receptor expression patterns in the hippocampus of male and female mice [18], and in male mice [33,38]. Several novel advances are conferred by the current work using a genetic model combined with high-resolution through-focus confocal microscopy. First, the improved resolution of D2 receptor expression allowed for quantitative analysis of hippocampal subregions not previously examined, including CA1, CA3, FC, and lacunosum moleculare. Second, the inclusion of female mice enabled the finding that female sham mice have lower neuronal D2 expression in the hippocampus compared to age-matched sham males. Third, the results elucidate the interaction effects between FPI and biological sex. These results are discussed in the context of neuroanatomical, physiological, and functional manifestations.

In the current study, FPI induced a significant loss of D2-expressing cells in the dentate gyrus of male mice. These reductions included the hilus, GCL, and OML. Additionally, there was a significant loss of D2-expressing neurons within the lacunosum moleculare and a significant decrease in D2-expressing axonal boutons in the CA3 in male FPI mice compared to male sham mice (Figure 3). However, female mice lacked any such significant reductions in D2-expressing neurons following FPI and, in some cases, exhibited a modest, albeit non-significant, increase in expression (Figure 4). The observation that female mice are more resilient to D2 neuroplasticity following an FPI is consistent with previous studies demonstrating that female mice are more resistant to TBI-induced neurodegeneration and neuroplasticity [58,59,60,61,62,63,64,65,66,67]. In the hippocampus, dopamine integrates spatial learning and memory with the dopaminergic reward system. As such, there may be functional implications for the differences in hippocampal D2 neuroplasticity observed in females and males in response to FPI.

Hippocampal dopaminergic circuitry is involved in focusing attention on novel and salient information and helps to regulate synaptic plasticity [68]. With regard to the spatial component of hippocampal processing, exposure to novel environments induces the hippocampus to signal the midbrain ventral tegmental area (VTA) via the nucleus accumbens and ventral pallidum. Activation of this circuitry results in hippocampal dopamine release that contributes to encoding place into the memory engram [69,70]. Hippocampal D2-like receptors mediate bi-directional synaptic plasticity that is important for spatial learning [71], and D2 receptors within the pyramidal cell layer enhance the consolidation of novel information into long-term memories, possibly via actions on the limbic reward system pathway [72]. In addition, D2-receptors in the hippocampus modulate reward-associated learning and long-term memory consolidation associated with reward-seeking behaviors [73]. Therefore, hippocampal dopaminergic signaling and its link between place, spatial memory, and the reward circuit are critically involved in reward-seeking behavior. The functional implications of differences in D2 expression between males and females and in the neuroplastic response of D2 receptors to a TBI remain to be elucidated, but available evidence suggests that they may influence reward circuitry.

TBIs are known to induce increased drug and alcohol-seeking behavior. These behaviors are known to involve the reward circuitry, and studies involving CPP provide a window into the importance of the hippocampal dopaminergic circuitry in reward-seeking behaviors. Dopaminergic reinforcement drives the circuitry involved in CPP [26,74,75]. In particular, D2 antagonism in the dorsal hippocampus can suppress CPP [73,76], and D2 signaling plays a role in CPP involving exposure to nicotine, cannabinoids, and drugs of abuse [77,78]. Moreover, D2 antagonism in the hippocampus can suppress morphine-associated CPP [73,76,79]. Interestingly, dopaminergic signaling in the dorsal hippocampus is also involved in state-dependent learning in response to several drugs of abuse [77,78,80]. Therefore, hippocampal dopaminergic signaling, notably via D2 receptors, is an important component in reinforcement learning and memory. Alterations to this hippocampal reward circuit after TBI might contribute to changes to drug and alcohol-seeking behaviors that are often observed after injury [81,82,83].

The influence of biological sex after TBI is epitomized by the observation that males and females have different rates of post-traumatic drug and alcohol disorders [4,84,85]. Consistent with these clinical data, males and females perform differently in CPP after TBI [31,32,86]. Females are more susceptible to addiction and drug-related effects than males [87], both in humans [88,89] and in rodent models [90,91]. Females are also more vulnerable to the drug-associated effect on CPP for a number of drugs of abuse, including mephedrone [92], methamphetamine [93], cocaine [90,94], and nicotine [95]. It is possible that the previously reported differences in CPP performance after TBI [28,29] are related to the interactions between sex and injury on D2-expressing neurons. The synaptic strength of the hippocampal–nucleus accumbens circuitry regulates rewarding behavior, and induction of LTP at this synapse drives CPP in response to rewarding interactions [96]. Unfortunately, a limitation of the current study, in addition to the low n in males, is that we did not assess dopamine changes or receptor sensitivity in the hippocampus before or after injury. Future studies are needed to fully understand the functional consequences of reduced D2 receptors in females compared to males and the influence of TBI on the plasticity of the hippocampal dopaminergic system.

## 4. Materials and Methods

### 4.1. Animals

Eight-week-old male and female transgenic D2-Cre;Ai14-tdTomato mice were obtained from our breeding facility. All experimental procedures were conducted in accordance with the Institutional Animal Care Committee (IACUC) of the Texas A&M Health Science Center guidelines. The mice were housed at the Texas A&M Health Science Center animal facility, provided with food and water ad libitum, and maintained on a 12:12 light/dark cycle. Animals were divided into four total treatment groups for all analyses: Male-Sham (n = 2), Male-FPI (n = 2), Female-Sham (n = 3), and Female-FPI (n = 3). While these animal numbers were relatively low, particularly in the male groups, we observed a large effect of FPI on D2 expression (Cohen’s d > 1). Still, it is important to recognize this potential limitation of the study.

### 4.2. Fluid Percussion Injury (FPI)

At nine weeks of age, tdTomato D2 transgenic mice underwent either a sham injury or a lateral fluid percussion injury (FPI), as previously described [40,41,97]. Briefly, mice were anesthetized and placed within a stereotaxic instrument for surgery (Stoelting, Wood Dale, IL, USA). A cranial incision was made to expose the skull. The animals in the sham group experienced only a sterile craniotomy, while the TBI group underwent a 2 mm craniotomy and a subsequent lateral FPI. The FPI was administered at 1.5 posterior and 1.2 medial to the bregma point. The injury consisted of a brief (12–16 ms) pressure pulse (~1.2–1.6 atm) from the FPI apparatus (Custom Design & Fabrication, Richmond, VA, USA).

### 4.3. Tissue Preparation

At 4 weeks after TBI or sham, the mice were sacrificed and underwent transcardial perfusion using sterile saline until the blood ran clear, followed by 4% paraformaldehyde (PFA), as previously described [98]. Following the perfusion, the brains were allowed to post-fix in PFA in the skull for 24 h, after which the brains were extracted and placed in a 4% PFA solution for an additional 48 h. The brains were then cryoprotected in sucrose solution and placed in phosphate buffer solution (PBS) until they were cut. A cryostat was used to serially section the brains in the coronal plane at 50-micron intervals. The tissue slices were placed in 12-well plates filled with PBS and kept at 4 °C until mounting was conducted. Slices of tissue with the hippocampal regions of interest were selected. Mounting of these coronal slices on charged glass slides was performed, after which coverslips were applied, and imaging was conducted through the use of a confocal microscope.

### 4.4. Confocal Imaging and Stitching

Automated confocal z-stack images were created, and subsequent stitching of the confocal images was performed using Fluoview software (Version 4.2; Olympus; Center Valley, PA, USA). Briefly, slides containing the hippocampus were aligned under the confocal microscope, and each hippocampus was traced. The confocal microscope (Olympus; Center Valley, PA, USA) was set to automatically scan through the x, y, and z planes. Once the large image files were obtained, they were then loaded into software (Imaris, Bitplane Version 4.2; Belfast, UK) and stitched to create an entire composite image of each hippocampus (Figure 1).

### 4.5. Qualitative Analysis and Quantification of D2-Expressing Neurons

Qualitative analysis consisted of viewing multiple image stacks by blinded reviewers. Using the native image stacks (Figure 1), region-by-region descriptions of the appearance of D2-expressing neurons were assessed. Quantitative analysis occurred with the rater blind to the treatment condition and sex of the mice. To quantify D2-expressing neurons and synaptic boutons, a manual counting regimen was used. Each region of the dorsal hippocampus that fell within the range of −1.46 to −2.06 mm bregma was assigned a specific color, and Photoshop (Version 24.x; Adobe; San Jose, CA, USA) was utilized to manually count the cells within each region. Only brightness and contrast were adjusted for the images. Serial sections were cut and slices within the dorsal hippocampus were selected for qualitative and quantitative analysis. Fifteen anatomical brain regions were quantified in total, including the three layers of the dentate gyrus (hilus, granular cell layer/subgranular zone (GCL), and molecular layer), hippocampal CA1, CA2, and CA3, which were examined by subfields (stratum oriens, stratum pyramidales, and stratum radiatum), as well as the fasciola cinereum and lacunosum moleculare. Counting in the CA2 included the area Ramon y Cajal described as stratum lacunosum subjacent to the CA2 stratum radiatum [99,100,101]. Axonal boutons that extend within the CA2 and CA3 pyramidal layers were also quantified. The cells of the inner molecular layer were not counted due to their robust density. However, cells of the outer molecular layer were quantified. Cells in the granule cell layer closer to the hilus were also quantified. The data were quantified bilaterally, and the average number of cell counts per slice, per hemisphere, from at least three slices per mouse was used for quantitative analysis.

### 4.6. Statistical Analysis

Analyses for males versus females after FPI were performed by two-way analysis of variance (ANOVA), with comparisons between groups performed using post hoc Bonferroni’s multiple comparison test. An unpaired Student’s *t*-test was used to compare male versus female sham (baseline) and sham versus FPI within the sexes. All statistical analysis was performed using GraphPad Prism (Version 9.0; GraphPad; San Diego, CA, USA). Significance for all tests was set at *p* < 0.05, and a trend was considered when *p* < 0.10.

## 5. Conclusions

In conclusion, the results from the current study identified, for the first time, sex-related differences in the expression of D2 receptors in the hippocampus, with females in general having reduced expression compared to males. These results additionally highlight that TBI-induced reductions in D2 receptor expression were much greater in males than in females, most notably in the dentate gyrus. These findings are consistent with previous studies showing that female rodents are generally more resilient to the neurodegenerative effects of TBI and may shed light on the neurochemical basis for sex differences in addiction and related behavioral phenomena, particularly after TBI [102]. Further investigation is warranted to better understand the mechanisms responsible for the observed sex-related differences and to investigate the functional consequences of differential regulation of D2 receptor expression following a TBI.

## Figures and Tables

**Table 1:** Effects of biological sex and FPI on D2-expression in the ipsilateral hippocampus. Two-way ANOVA was used to assess the effect of sex, FPI, and their interaction on the expression of D2 in the ipsilateral hippocampus. Post hoc analysis was conducted to investigate differences between males and females within each group. Significant effects or interactions are highlighted in yellow (*p* < 0.05), and trends (0.05 < *p* < 0.10) are highlighted in orange. The most prominent effects were seen in the CA2 axonal boutons, including a significant effect of sex, FPI, and interaction, as well as significantly less boutons in female sham animals compared to males. There are also robust differences found in the dentate gyrus, including a significant interaction effect in all three dentate gyrus regions (hilus, granule cell layer, and outer molecular layer). All three dentate regions also exhibit significant differences between male and female FPI groups, indicating increased D2 expression in female FPI animals. A significant interaction was also identified in the fasciola cinereum. Data are represented as mean ± SEM, n = 2 for male groups, and n = 3 for female groups. * *p* < 0.05, ** *p* < 0.01.

Region of Interest		2-Way ANOVA (*p*-Value)			Sham			FPI		
		Sex	FPI	Interaction	Male	Female	*p*-Value	Male	Female	*p*-Value
**CA1**	Stratum Radiatum	0.9814	0.8497	0.1994	33.00 ± 5.66	20.33 ± 4.75	0.6795	22.25 ± 4.60	34.50 ± 22.10	0.7096
	Stratum Oriens	0.3535	0.6012	0.1967	74.75 ± 19.45	84.67 ± 55.23	>0.9999	94.75 ± 1.77	40.17 ± 17.61	0.2658
**CA2**	Stratum Lacunosum	0.9084	0.5281	0.9948	15.17 ± 4.48	14.50 ± 9.73	>0.9999	11.25 ± 0.35	10.5 ± 12.13	>0.9999
	Stratum Radiatum	0.2163	0.1665	0.1775	47.58 ± 53.62	6.00 ± 3.12	0.1708	3.250 ± 2.475	5.333 ± 4.509	>0.9999
	Stratum Oriens	0.7386	0.8408	0.911	8.667 ± 0.943	7.000 ± 2.646	>0.9999	7.500 ± 2.121	6.667 ± 9.074	>0.9999
	Axonal Boutons	0.0223 *	0.0102 *	0.0193 *	63.83 ± 28.52	10.00 ± 7.55	0.0091 **	4.50 ± 3.54	5.50 ± 8.23	>0.9999
**CA3**	Stratum Radiatum	0.1475	0.4212	0.4036	10.75 ± 1.06	16.17 ± 11.64	>0.9999	10.50 ± 3.54	28.67 ± 14.84	0.2403
	Stratum Pyramidales	0.5769	0.9964	0.9445	26.67 ± 6.13	32.33 ± 10.61	>0.9999	27.25 ± 16.62	31.67 ± 16.01	>0.9999
	Stratum Oriens	0.0823	0.2639	0.6432	24.17 ± 9.66	16.67 ± 4.91	0.6042	32.250 ± 2.475	20.170 ± 9.224	0.2379
	Axonal Boutons	0.5135	0.1587	0.0698	256.8 ± 25.8	161.0 ± 50.6	0.654	14.75 ± 9.55	198.7 ± 161.50	0.1728
**DG**	Hilus	0.2197	0.1445	0.0042 **	126.3 ± 23.6	93.0 ± 7.1	0.142	60.25 ± 3.18	123.0 ± 22.34	0.0123 *
	Outer Molecular Layer	0.0091 **	0.0106 *	0.0145 *	117.8 ± 27.9	124.2 ± 31.4	>0.9999	2.25 ± 1.768	120.0 ± 23.52	0.0045 **
	Granule Cell Layer	0.2814	0.0253 *	0.0065 **	94.33 ± 11.79	65.83 ± 19.78	0.1739	25.00 ± 0.71	76.83 ± 15.45	0.0198 *
Lacunosum Moleculare		0.0369 *	0.8459	0.4168	100.50 ± 3.54	86.17 ± 19.66	0.9521	53.25 ± 1.77	62.17 ± 29.76	>0.9999
Fasciola Cinereaum		0.9437	0.1041	0.0138 *	23.92 ± 7.66	12.50 ± 4.77	0.1098	5.75 ± 1.06	17.67 ± 5.51	0.0953

**Table 2:** Effects of biological sex and FPI on D2-expression in the contralateral ippocampus. Two-way ANOVA was used to assess the effect of sex, FPI, and their interaction on the expression of D2 in the contralateral hippocampus. Post hoc analysis was conducted to investigate differences between males and females within each group. Significant effects (*p* < 0.05) or interactions are highlighted in yellow, and trends (0.05 < *p* < 0.10) are highlighted in orange. Overall, there were fewer significant differences identified in the contralateral hemisphere compared to the ipsilateral. However, there were significant effects of sex in both the CA1 stratum oriens, CA3 stratum radiatum, and dentate gyrus outer molecular layers. There were no significant FPI or interaction effects in the contralateral hemisphere. Data are represented as mean ± SEM, n = 2 for male groups, and n = 3 for female groups. * *p* < 0.05.

Region of Interest		2-Way ANOVA (*p*-Value)			Sham			FPI		
		Sex	FPI	Interaction	Male	Female	*p*-Value	Male	Female	*p*-Value
**CA1**	Stratum Pyramidales	0.9881	0.0774	0.4065	27.50 ± 2.12	25.17 ± 2.36	>0.9999	19.75 ± 1.06	22.00 ± 6.25	>0.9999
	Stratum Oriens	0.0370 *	0.1062	0.8009	80.92 ± 9.31	47.80 ± 11.88	0.1666	56.50 ± 27.58	29.33 ± 18.77	0.2792
**CA2**	Stratum Radiatum	0.1302	0.2816	0.4146	19.33 ± 3.30	9.333 ± 3.329	0.2248	11.50 ± 8.485	8.167 ± 7.182	>0.9999
	Stratum Pyramidales	0.4563	0.153	0.7299	11.00 ± 8.485	7.667 ± 1.443	0.8884	5.25 ± 1.061	4.00 ± 4.583	>0.9999
	Stratum Oriens	0.3257	0.4102	0.7978	12.420 ± 7.189	7.00 ± 4.583	0.7613	7.750 ± 4.596	4.50 ± 7.794	>0.9999
	Axonal Boutons	0.5168	0.9276	0.3217	3.50 ± 2.212	25.33 ± 20.64	0.5153	18.00 ± 24.75	13.17 ± 19.04	>0.9999
**CA3**	Stratum Lacunosum	0.2444	0.8637	0.7128	24.17 ± 1.18	16.17 ± 6.11	>0.9999	26.00 ± 31.11	11.17 ± 6.45	0.5614
	Stratum Radiatum	0.0243 *	0.6683	0.6904	52.08 ± 2.00	25.67 ± 5.53	0.2379	61.00 ± 33.23	26.00 ± 13.23	0.1053
	Stratum Oriens	0.1871	0.284	0.4851	28.50 ± 13.44	7.833 ± 2.754	>0.9999	81.750 ± 102.900	19.830 ± 12.090	0.331
	Axonal Boutons	0.1022	0.5599	0.4632	314.7 ± 79.7	151.7 ± 67.6	0.2074	230.50 ± 98.99	161.70 ± 115.70	0.8986
**DG**	Hilus	0.4841	0.8398	0.1856	108.7 ± 6.13	99.17 ± 25.48	>0.9999	87.0 ± 12.02	115.5 ± 20.62	0.3286
	Outer Molecular Layer	0.028 *	0.8294	0.9541	135.0 ± 25.46	86.83 ± 11.54	0.1647	132.3 ± 37.12	82.17 ± 27.9	0.1465
	Granule Cell Layer	0.4008	0.8435	0.735	91.25 ± 10.96	72.17 ± 28.07	0.8155	89.0 ± 23.33	80.67 ± 23.12	>0.9999
Lacunosum Moleculare		0.4757	0.2408	0.6632	91.5 ± 0.71	88.00 ± 19.49	>0.9999	81.75 ± 8.84	67.67 ± 23.29	0.8441
Fasciola Cinereum		0.188	0.4613	0.5665	24.42 ± 8.37	16.67 ± 6.53	0.3804	19.25 ± 1.06	16.00 ± 4.58	>0.9999

## Data Availability

The data for the current study will be made available upon reasonable request by the corresponding authors.
